# Association of IL-6 and IL-17 with thyroid eye disease

**DOI:** 10.3389/fendo.2026.1798278

**Published:** 2026-05-08

**Authors:** Xin Sun, Yanhua Jiang, Zhili Liu, Lei Zhao

**Affiliations:** 1 Department of Endocrinology, First Affiliated Hospital of Soochow University, Suzhou, China; 2 Department of Ophthalmology, Fourth People`s Hospital of Shenyang, Shenyang, China; 3 Department of Ophthalmology, Second Affiliated Hospital of Liaoning University of Traditional Chinese Medicine, Shenyang, China

**Keywords:** IL-17, IL-6, meta, TED, thyroid eye disease

## Abstract

**Background:**

Thyroid Eye Disease (TED) is the most common extrathyroidal manifestation of Graves’ disease. Interleukin-6 (IL-6) and interleukin-17 (IL-17) have been implicated in its pathogenesis, but individual study results remain inconsistent. However, existing findings on IL-6 and IL-17 levels in the serum or tears of TED patients remain inconsistent.

**Aim:**

This meta-analysis aimed to statistically evaluate the level of IL-6 and IL-17 in patients with TED.

**Methods:**

A systematic literature search was conducted across five electronic databases (PubMed, Web of Science, Elsevier Science Direct, Wiley Online Library, and CNKI). The search strategy targeted the terms “Thyroid Eye Disease” in conjunction with “Interleukin-6” or “Interleukin-17” in title and abstract fields. Results are presented as standardized mean differences (SMD) with 95% confidence intervals (CI).

**Results:**

This meta-analysis demonstrated significantly elevated levels of both IL-6 and IL-17 in patients with TED compared to controls. Pooled estimates showed a substantial increase in IL-6 (SMD: 1.68; 95% CI: 0.83–2.53), with substantial heterogeneity. Similarly, IL-17 levels were markedly higher in TED patients overall (SMD: 1.78; 95% CI: 1.18–2.37). Furthermore, patients with active TED exhibited significantly higher IL-6 and IL-17 levels than those with inactive disease.

**Conclusions:**

To our knowledge, this meta-analysis is a large-scale systematic evaluation of IL-6 and IL-17 levels in patients with TED. These findings describe the circulating cytokine profile in the studied population and may inform future hypothesis-driven research.

**Systematic Review Registration:**

https://www.crd.york.ac.uk/PROSPERO/view/CRD420261277844, identifier CRD420261277844.

## Introduction

Thyroid Eye Disease (TED), also known as Graves’ Orbitopathy, is the most common extrathyroidal manifestation of Graves’ disease, characterized by orbital inflammation, tissue remodeling, and potential vision loss (1). Approximately 25–50% of Graves’ disease patients are affected by TED, and 3–5% may develop severe eye disease or even progressive vision loss. Its pathogenesis involves a complex interplay of autoantibodies, particularly against the TSH receptor (TSHR), and a cascade of inflammatory cytokines that drive adipogenesis, glycosaminoglycan deposition, and fibrosis within the orbit (2).

Emerging evidence highlights that this autoimmune-driven orbital pathology is sustained not only by TSHR-stimulating antibodies but also by a dysregulated cytokine milieu in which specific pro-inflammatory mediators act both as effectors and amplifiers of local immune responses (3). Among these, interleukin-6 (IL-6) and IL-17 have emerged as a central node linking autoimmunity, fibroblast activation, and chronic inflammation in the orbital microenvironment (4). Production of IL-6 is regulated by several cell types involved in TED, including fibrocytes, orbital fibroblasts (OFs), adipocytes, and orbital macrophages, and is also the result of OF-lymphocyte crosstalk. Studies suggest that IL-6 drives TED pathogenesis through multiple mechanisms that together may contribute to inflammation, tissue expansion, remodeling, and fibrosis within the orbit (5). IL-6 also promotes the differentiation of B cells and Th17 cells, linking innate and adaptive immunity. IL-17, primarily produced by Th17 cells, is a potent pro-inflammatory cytokine implicated in various autoimmune diseases. It can synergize with other cytokines to amplify inflammation and is thought to contribute to the tissue remodeling phase of TED (6).

Despite numerous individual studies measuring IL-6 and IL-17 in serum or tears of TED patients, findings have been inconsistent. Some studies report significant elevation (7, 8), while others show no difference or even lower levels compared to controls (9, 10). This inconsistency may stem from variations in study design, sample types, disease phase, and treatment status. Therefore, this systematic review and meta-analysis is to synthesize existing evidence on the association of IL-6 and IL-17 levels with TED.

## Methods

### Search

Comprehensive searches were conducted in the following electronic databases: PubMed, Web of Science, Elsevier Science Direct, Wiley Online Library, and CNKI. The search combined MeSH terms and keywords: (“Thyroid Eye Disease” OR “Graves Orbitopathy” OR “Thyroid-associated Ophthalmopathy”) AND (“Interleukin-6” OR “Interleukin-17”). All relevant publications from 1980 through 2025 were considered. The complete electronic search strategy, including keywords and database-specific syntax, was provided in the **Supplementary File S1**. In addition, reference lists of the retrieved articles were reviewed to identify other potentially eligible studies; however, unpublished reports were excluded. A completed PRISMA checklist is available in the **Supplementary File S2**. The protocol for this systematic review and meta-analysis was registered with PROSPERO (registration number: CRD420261277844).

### Inclusion criteria

Studies were included in the meta-analysis if they met the following criteria: (1) Only case-control studies and cohort studies were included. Cross-sectional studies with appropriate control groups were also considered if they met the eligibility criteria; (2) the studies reported tear or serum of IL-6 and IL-17 levels in patients with TED and healthy control groups; (3) the studies were published in English and Chinese due to the language proficiency of the authors and to ensure a practical and consistent screening of potentially relevant studies.

### Exclusion criteria

Studies were excluded based on the following criteria: unavailability of the full text, duplication of publications, incomplete or non-convertible data, and the implementation of interventions in either the experimental or control groups that did not comply with the study protocol. Furthermore, exclusion applied to studies with significant methodological flaws, as well as non-human research, review articles, conference abstracts, case reports, and editorial commentaries.

### Data extraction and risk of bias

Two investigators independently performed literature screening, data extraction, and cross-validation. Any discrepancies were resolved through discussion or by consulting a third reviewer. Screening began with a review of article titles, followed by the exclusion of irrelevant studies. Subsequently, abstracts and full texts of the remaining articles were examined to assess eligibility for inclusion. When necessary, corresponding authors were contacted via email to obtain missing data. The extracted information included the title, first author, year of publication, study location, sample size, participant age per group, and relevant outcome indicators.

The Newcastle–Ottawa Scale (NOS), which was recommended by the Cochrane Collaboration for assessing the quality of observational studies, was used to evaluate risk of bias (11, 12). Each included study was judged according to 3 domains using the “star system”: representativeness of study group selection (4 items), comparability of groups (two items), and ascertainment of either the exposure or outcome (3 items). NOS scores range from 0 to 9 stars. Two researchers independently rated each study, compared scores, and resolved inconsistencies through consensus. In cases where agreement could not be reached, a third researcher was consulted to make the final determination.

### Statistical analysis

Given the heterogeneity in measurement units and detection platforms across studies, the standardized mean difference (SMD) was chosen as the effect size metric to enable pooling of data on a common scale. The results of this meta-analysis are expressed as SMDs with 95% confidence intervals (CIs). For studies reporting median (interquartile range), we converted these to mean and standard deviation using the method described by Wan et al. (13). Specifically, we applied equation (3) for estimating the mean, and equations (9), (13), or (16) for estimating the standard deviation. As this study is a meta-analysis of existing literature, no *a priori* sample size calculation was performed; we included all eligible studies according to PRISMA guidelines. The final sample size was determined by data availability. Between-study heterogeneity was evaluated using Cochran’s Q test and the *I*² statistic. An *I*² value below 50% indicated low-to-moderate heterogeneity, in which case a fixed-effect model was applied; otherwise, a random-effect model was used. Sensitivity analysis was conducted to examine the impact of individual studies on the overall results. Potential publication bias was assessed using Begg’s and Egger’s tests, where a *P*-value of <0.05 was considered statistically significant. All analyses were performed using Stata version 12.0 (StataCorp, College Station, TX, USA).

## Results

**Figure 1:**
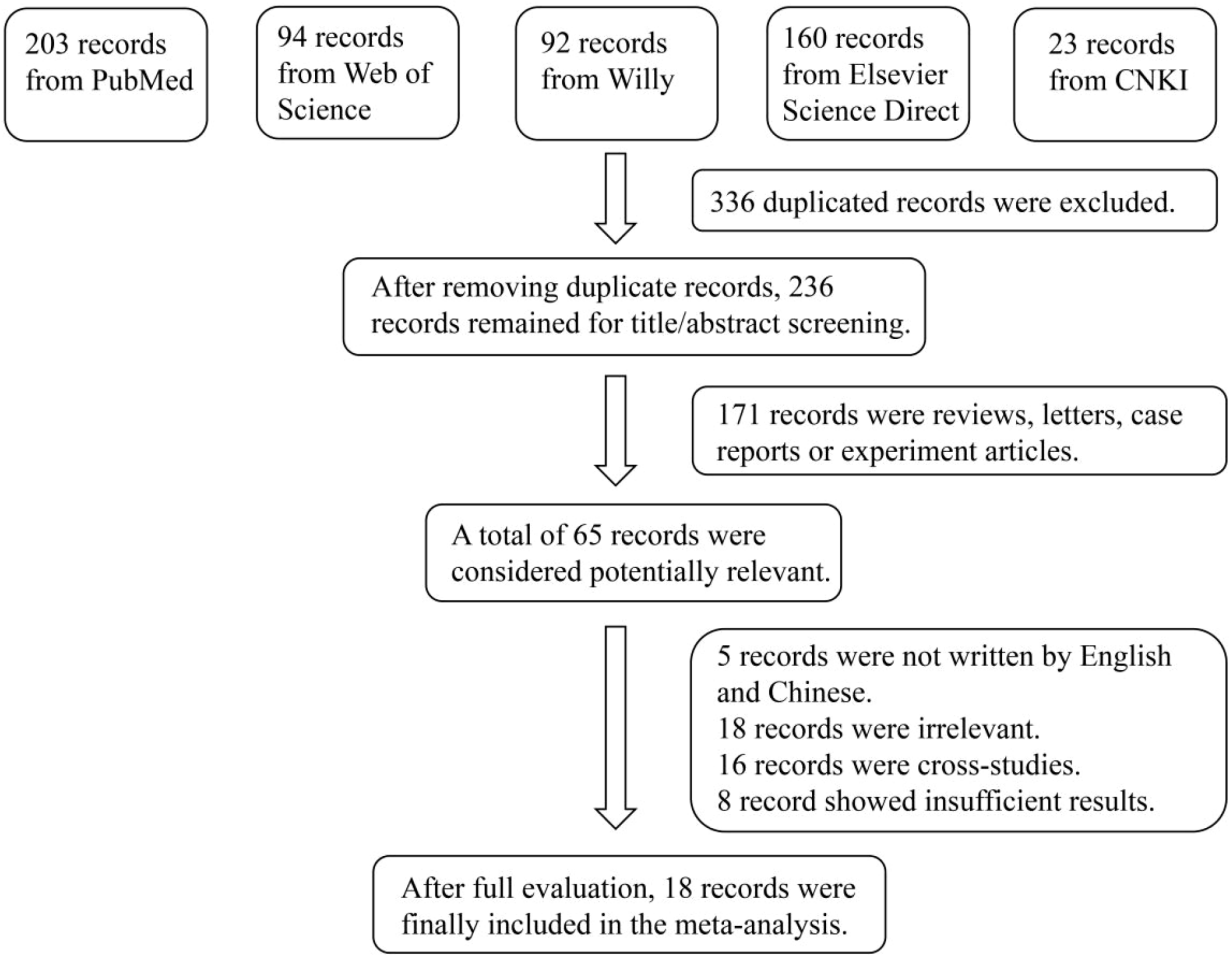
Flowchart of the detailed procedure for the inclusion or exclusion of selected studies.

A total of 572 records were initially identified. After removing 336 duplicates, 236 records were screened by title and abstract. Of these, 171 records were excluded as irrelevant, leaving 65 full-text articles assessed for eligibility. Finally, 18 articles comprising a total of 834 cases and 566 controls were included in the final meta-analysis (7–10, 14–27). The flowchart of the study selection process is shown in **Figure 1**, and the key characteristics of the included studies are presented in **Table 1**.

**Table 1:** Study characteristics of the published studies included in the meta-analysis.

Author	Year	Region	Number		Age(years)		Gender (Male/Female)		IL-6		IL-17		Sample	NOS score
			Case	Control	Case	Control	Case	Control	Case	Control	Case	Control		
Wakelkamp IM	2000	The Netherlands	62	62	53.1 ± 10.3	53.2 ± 9.7	19/43	19/43	3.16 ± 2.7	1.98 ± 2	–	–	Serum	7
Salvi M	2000	Italy	51	67	45 ± 1.0	48 ± 1.0	NR	6/61	3.2 ± 0.4	1.9 ± 0.2	–	–	Serum	7
Tang L	2002	China	50	30	42.54 ± 13.51	40 ± 10.22	27/23	16/14	94.45 ± 12.91	89 ± 14	–	–	Serum	6
Laban-Guceva N	2007	Macedonia	22	29	46.82 ± 12.47	51.86 ± 10.52	9/13	16/13	1.28 ± 0.92	1.72 ± 1.9	–	–	Serum	7
Kim SE	2012	Korea	62	19	39.8 ± 14.8	39.6 ± 16.5	14/48	6/13	1.28 ± 6.65	0.01 ± 0.0	8.4 ± 20.54	1.02 ± 4.38	Serum	7
Ujhelyi B	2012	Hungary	27	12	43.4 ± 15.2	38.6 ± 13.8	6/21	4/8	204 ± 106	119 ± 63.5	386 ± 266	179 ± 139	Tear	7
Wei H	2014	China	27	15	39.41 ± 11.98	44.3 ± 16.2	11/16	8/7	–	–	8094 ± 2556	1859.3 ± 438	Serum	8
Lv M	2014	China	30	15	NR	NR	NR	NR	15.98 ± 3.21	5.41 ± 0.87	13.57 ± 1.89	6.27 ± 0.84	Serum	6
Shen J	2015	China	45	10	39.0 ± 13.45	34.3 ± 12.2	23/22	5/5	6.8 ± 3.48	2.15 ± 0.94	34 ± 12.9	7.64 ± 3.28	Serum	6
Li W	2017	China	80	82	35.8 ± 4.75	35.9 ± 4.6	48/32	46/36	13.4 ± 7.8	5.1 ± 2	37.2 ± 15.3	15.7 ± 3.2	Tear	6
Wang Z	2017	China	67	30	42.63 ± 11.77	Age-matched	36/31	sex-matched	–	–	15.87 ± 4.39	6.17 ± 2.56	Serum	6
Li H	2018	China	46	22	44.25 ± 9.9	43.2 ± 9.7	14/32	6/16	–	–	1125 ± 240	582 ± 171	Serum	6
Zhang H	2019	China	54	30	39.39 ± 5.21	44.32 ± 6.21	22/32	16/14	–	–	12.75 ± 2.36	6.43 ± 1.06	Serum	6
Xu N	2020	China	15	15	43.19 ± 10.33	38.2 ± 12.64	3/12	4/11	113.37 ± 136.8	11.73 ± 7.56	9.56 ± 4.06	7.33 ± 2	Tear	7
Kosciuszko M	2021	Poland	15	10	47 (46–75)	58 (34–76)	2/13	3/7	–	–	22.4 ± 12.5	19.7 ± 11.7	Serum	6
Musakulova A	2023	Kazakhstan	51	17	43.36 ± 4.98	38 ± 5.4	24/27	12/5	–	–	Serum: 8.57 ± 6.3; Tear:28.4 ± 11	Serum: 0.21 ± 0.14; Tear:8.45 ± 1.9	Serum, Tear	7
Riguetto CM	2024	Brazil	33	16	53 ± 14.34	49.63 ± 12.48	8/25	4/12	–	–	3.57 ± 1.77	4.11 ± 0.7	Serum	7
Huang J	2024	China	63	63	37.27 ± 7.28	37.67 ± 7.52	31/32	31/32	151 ± 20	72 ± 12	–	–	Serum	6

NOS, Newcastle–Ottawa Scale; NR: Not reported.

### Results of the meta-analysis

**Figure 2:**
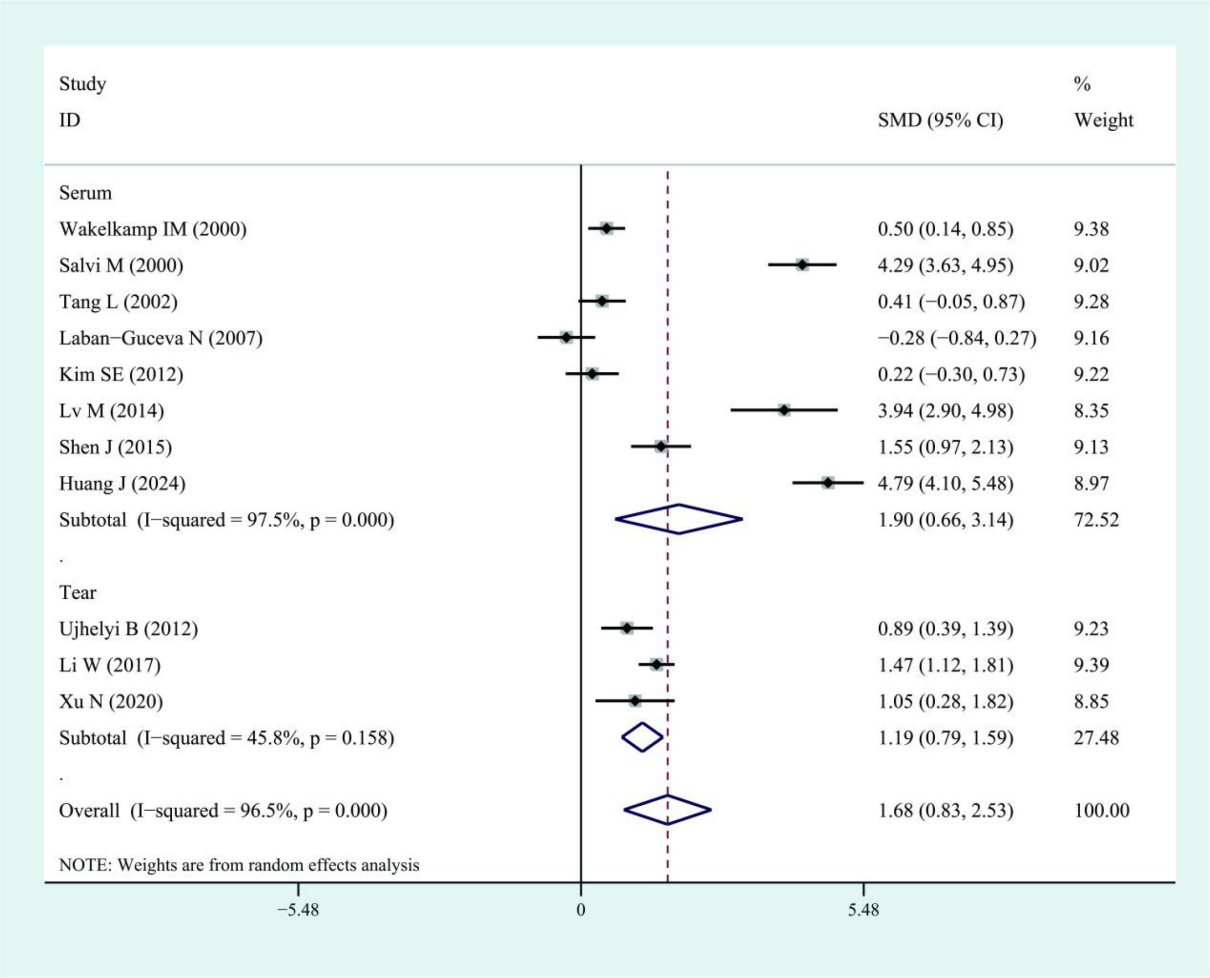
Forest plots of IL-6 in patients with thyroid eye disease compared to the control. Diamond represents the pooled SMDs at 95% CI. SMD, standardized mean difference; CI, confidence interval.

**Figure 3:**
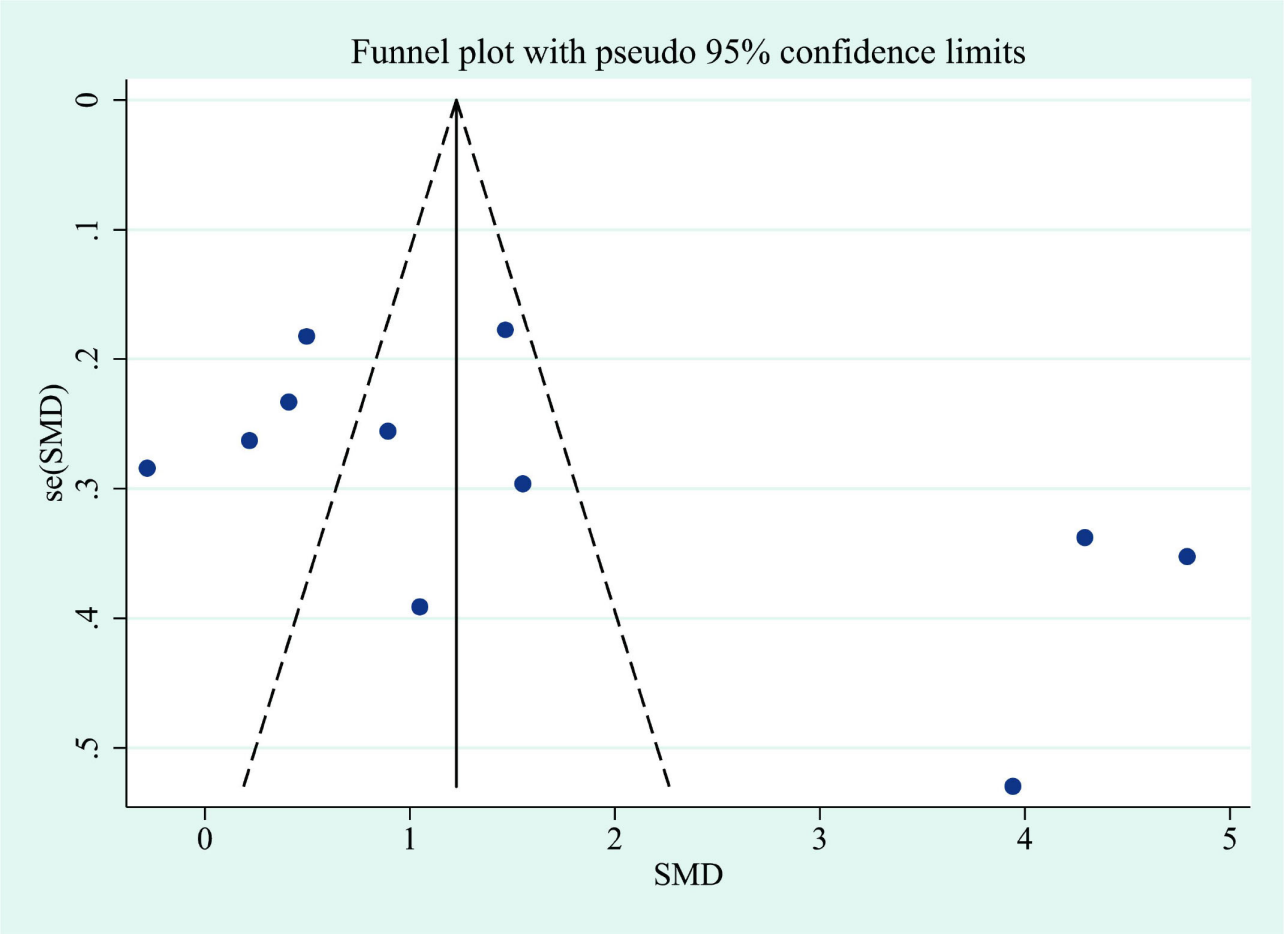
The funnel plots of IL-6 in patients with thyroid eye disease compared to the control. SMD, standardized mean difference.

**Figure 4:**
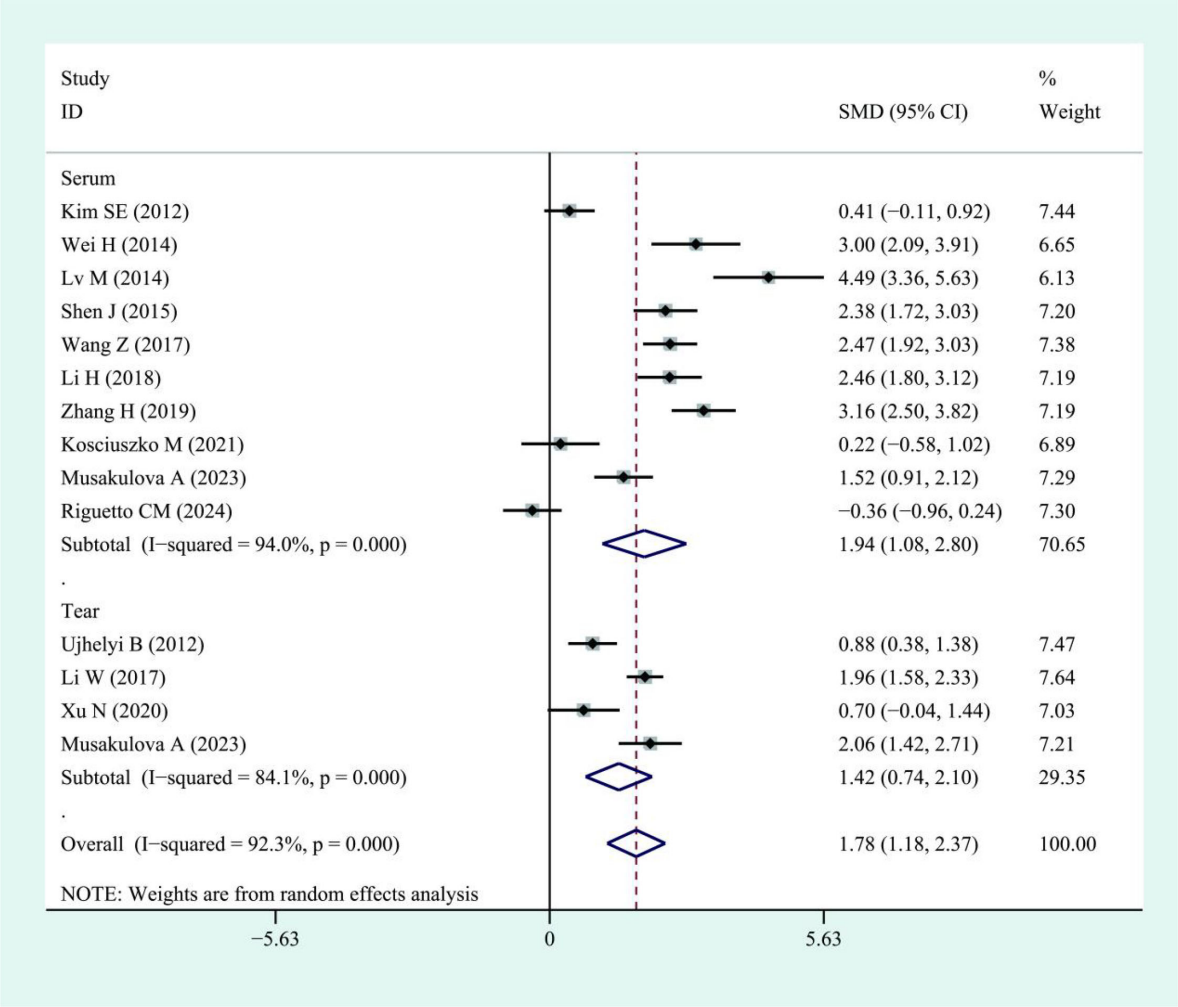
Forest plots of IL-17 in patients with thyroid eye disease compared to the control. Diamond represents the pooled SMDs at 95% CI. SMD, standardized mean difference; CI, confidence interval.

**Figure 5:**
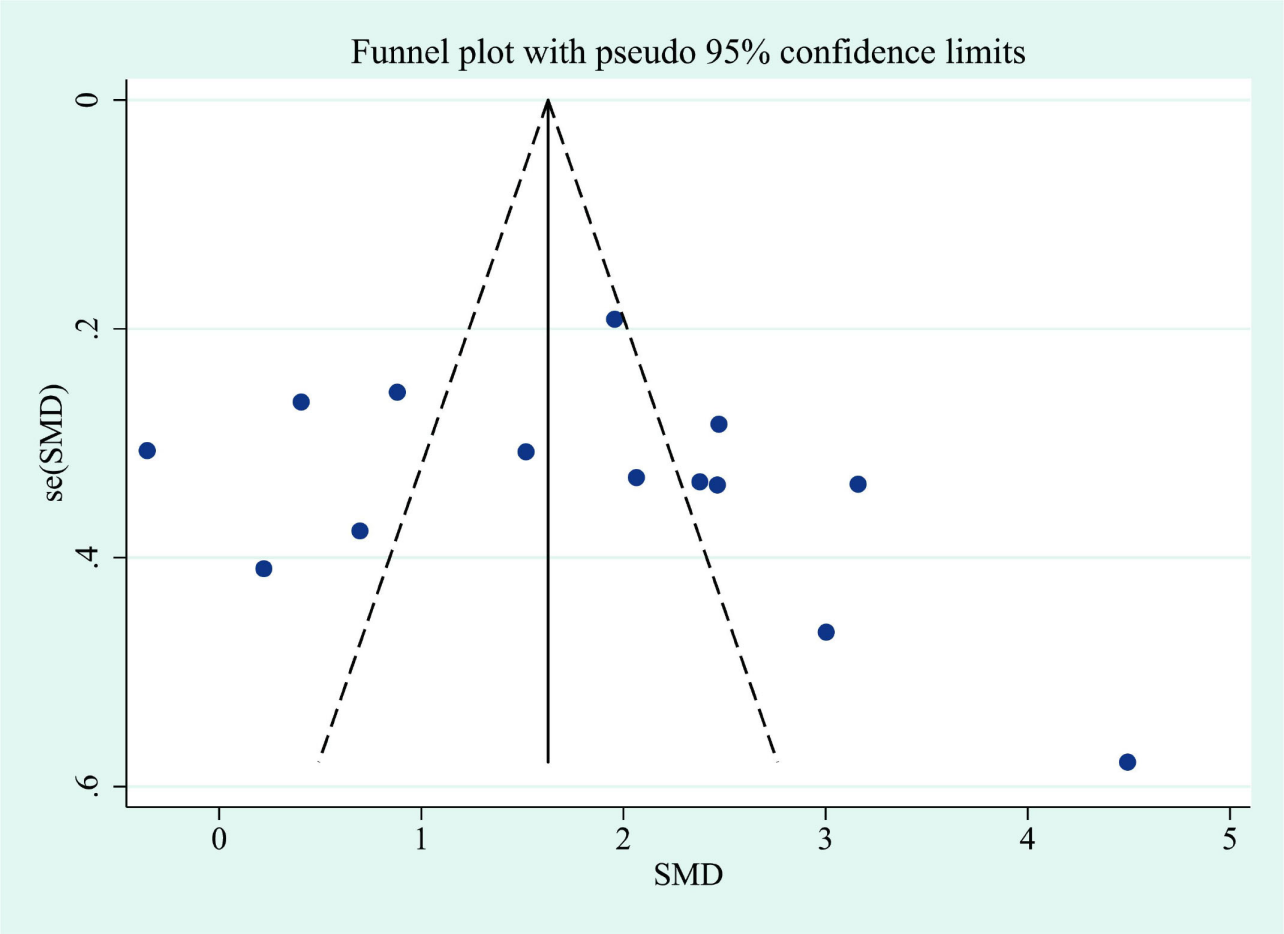
The funnel plots of IL-17 in patients with thyroid eye disease compared to the control. SMD, standardized mean difference.

This meta-analysis showed that IL-6 levels in TED patients were higher than those in controls, with a pooled SMD of 1.68 (95% CI: 0.83–2.53). However, substantial heterogeneity was observed across studies (I² = 96.5%). The corresponding forest plots and funnel plots were presented in **Figure 2** and **Figure 3**. Similarly, IL-17 levels in TED patients were higher relative to controls (SMD: 1.78; 95% CI: 1.18–2.37), with high heterogeneity (I² = 92.3%). The corresponding forest plots and funnel plots were presented in **Figure 4** and **Figure 5**.

To explore the potential sources of the substantial heterogeneity observed in the primary analyses, we performed meta-regression analyses. The following covariates were examined: publication year, geographic region, mean age of the case group, sample size, disease activity, and sample type. The results showed that publication year, geographic region, mean age, and sample size were not significantly associated with the observed heterogeneity. In contrast, disease activity and sample type were identified as significant contributors to heterogeneity (*P* < 0.05).

**Figure 6:**
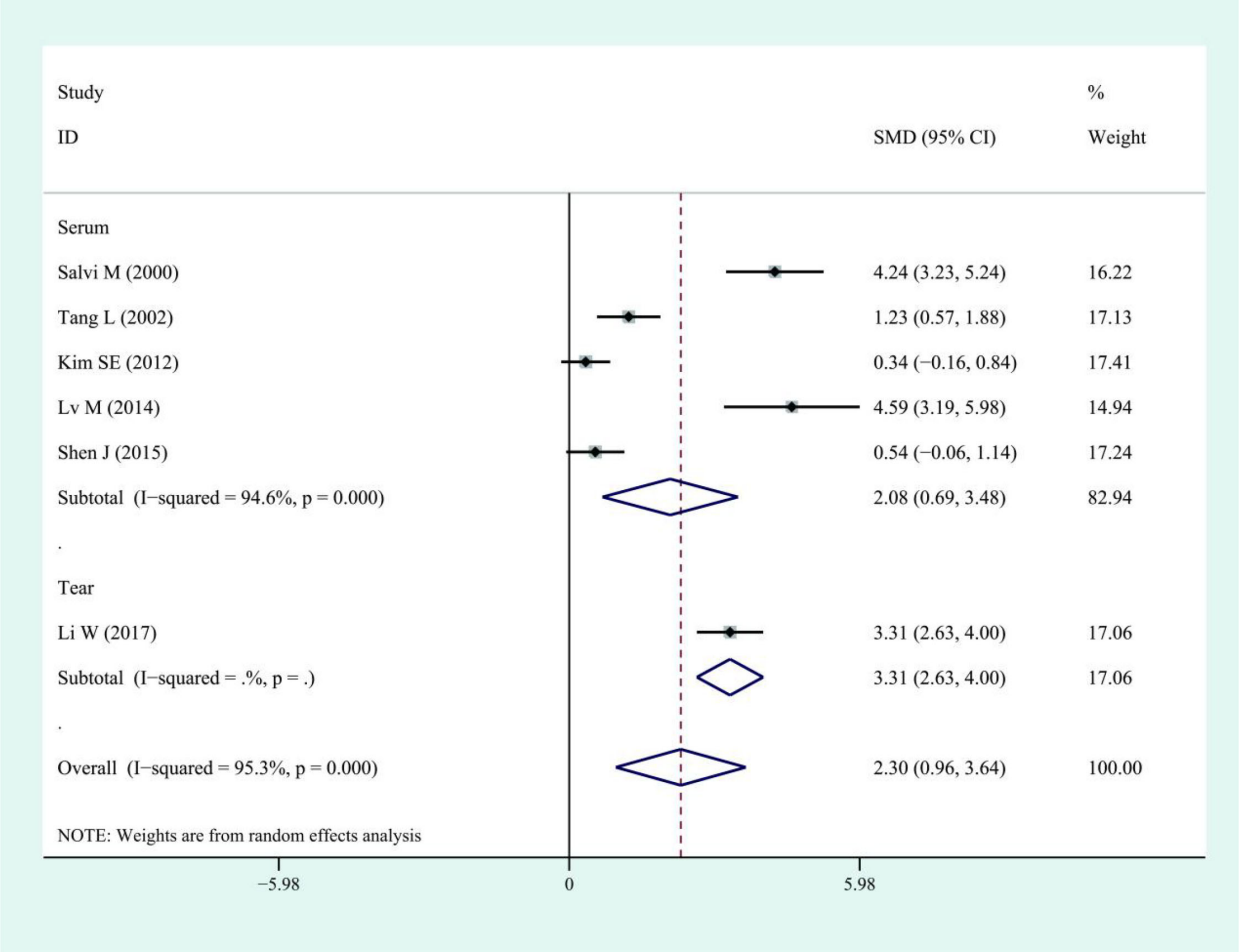
Forest plots of IL-6 in patients with active compared to inactive thyroid eye disease. Diamond represents the pooled SMDs at 95% CI. SMD, standardized mean difference; CI, confidence interval.

**Figure 7:**
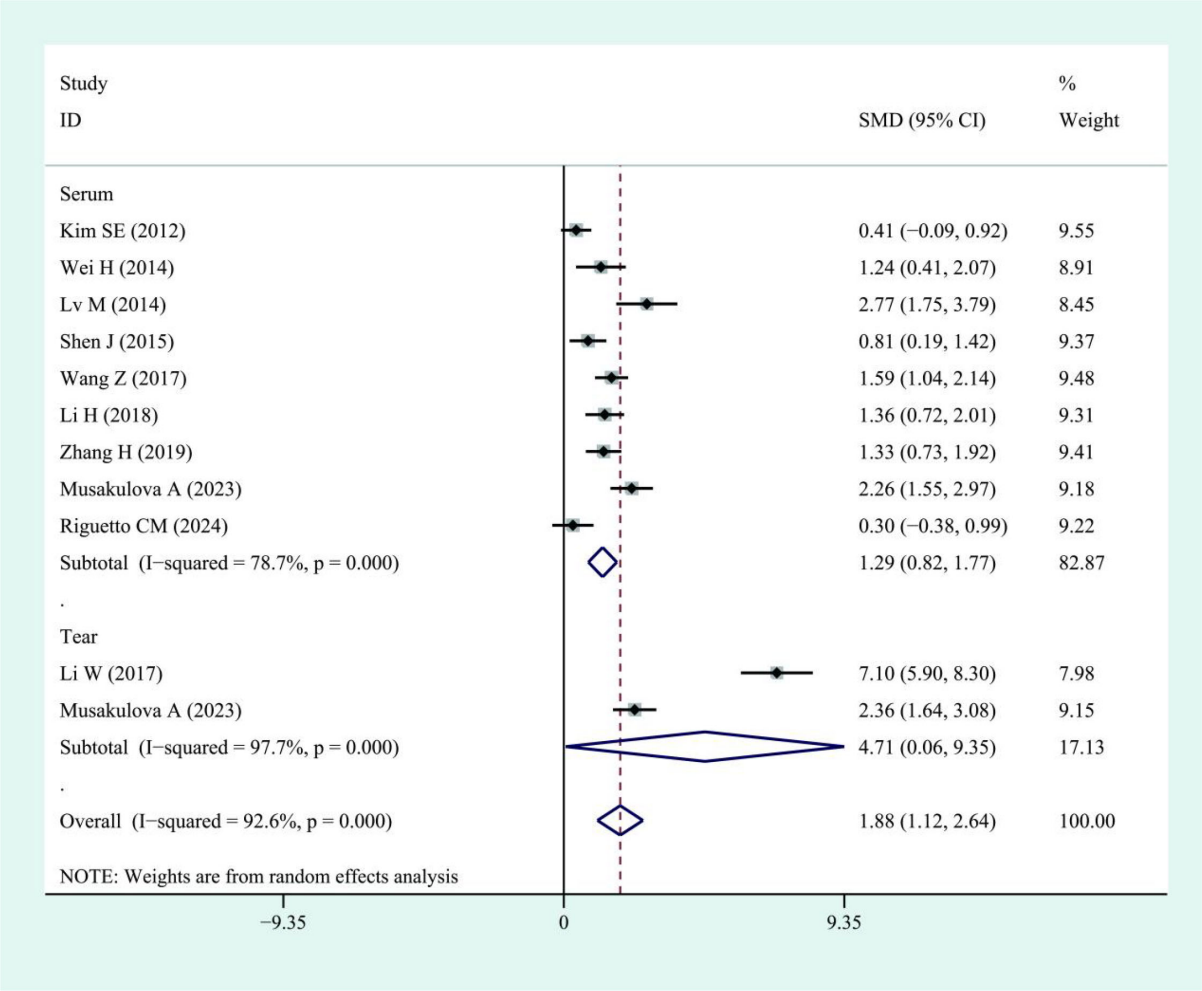
Forest plots of IL-17 in patients with active compared to inactive thyroid eye disease. Diamond represents the pooled SMDs at 95% CI. SMD, standardized mean difference; CI, confidence interval.

Subgroup analyses indicated higher IL-6 levels in both serum (SMD: 1.90; 95% CI: 0.66–3.14; I² = 97.5%) and tears (SMD: 1.19; 95% CI: 0.79–1.59; I² = 45.8%) of TED patients compared with controls. Similarly, IL-17 levels were elevated in TED patients compared with controls in both serum (SMD: 1.94; 95% CI: 1.08–2.80; I² = 94%) and tear samples (SMD: 1.42; 95% CI: 0.74–2.10; I² = 84.1%). Regarding disease activity, serum IL-6 levels were higher in patients with active TED than in those with inactive disease (SMD: 2.08; 95% CI: 0.69–3.48), although the heterogeneity was high (I² = 94.6%, **Figure 6**). Due to the limited sample size, findings on IL-6 levels in tear samples remained inconclusive. For IL-17, patients with active TED showed higher levels than those with inactive disease in both serum (SMD: 1.29; 95% CI: 0.82–1.77; I² = 78.7%) and tears (SMD: 4.71; 95% CI: 0.06–9.35; I² = 97.7%) (**Figure 7**).

### Sensitivity analysis and publication bias

The removal of any single study in the sensitivity analysis did not substantially alter the pooled effect size, confirming the robustness of our findings (**Supplementary Figures S1**, **2**, **3**, **4**). To assess potential publication bias, Begg’s and Egger’s statistical tests were performed following our comprehensive literature retrieval. Publication bias tests showed no significant bias for IL-6, as indicated by Begg’s test (continuity corrected P = 0.161) and Egger’s test (P = 0.105). Similarly, for IL-17, no significant publication bias was detected by Begg’s test (continuity corrected P = 0.155) or Egger’s test (P = 0.406).

## Discussion

To our knowledge, this meta-analysis is a large-scale systematic evaluation of IL-6 and IL-17 levels in patients with TED. Although previous studies have explored the association between IL-6, IL-17 and TED, their findings have been inconsistent. By synthesizing data from 18 independent studies, this analysis reveals that IL-6 and IL-17 levels are significantly elevated in patients with TED than in the control.

IL-6 is a pleiotropic cytokine produced by monocytes, macrophages, T lymphocytes, and other cell types, playing a central role in host defense against environmental stresses such as injury and infection. As a pleiotropic cytokine, IL-6 plays essential roles in immune regulation, inflammation, hematopoiesis, and metabolism, primarily involved in T-cell activation, B-cell differentiation into plasma cells, and the production of acute-phase proteins (28). As a key inflammatory factor in TED, IL-6 has been detected both in retro-orbital tissues of patients and in cultured orbital fibroblasts. It promotes abnormal expression of HLA class II antigens, leading to increased production of autoantibodies such as TSH receptor antibodies, stimulates thyroid hormone secretion, contributing to hyperthyroidism, and activates orbital fibroblasts to produce excessive extracellular matrix and collagen fibers, thereby driving disease progression (29).

Studies have shown elevated IL-6 levels and increased mRNA expression in thyroid cells of Graves’ disease patients using immunohistochemistry and *in situ* hybridization (30). Collier et al. (31) further confirmed high IL-6 expression in the extraocular muscles and orbital adipose tissues of TED patients, with a positive correlation between orbital volume and IL-6 mRNA expression.

IL-17, secreted mainly by TH17 cells, functions as a proinflammatory cytokine by inducing target cells to produce chemokines and colony-stimulating factors. It plays a role in promoting inflammatory cell infiltration and tissue destruction. Current research on IL-17 primarily focuses on its immunomodulatory effects. As a proinflammatory cytokine, IL-17 binds specifically to its receptor and activates NF-κB and mitogen-activated protein kinase pathways, leading to the expression of proinflammatory cytokines, monocyte chemotactic protein-1, intercellular adhesion molecules, and prostaglandin E2. These actions contribute to inflammatory cell infiltration, tissue injury, and the progression of inflammation and immune responses (32, 33).

In the field of targeted therapy for TED, teprotumumab—a monoclonal antibody against the insulin-like growth factor-1 receptor (IGF-1R)—has become a widely used treatment. Meanwhile, tocilizumab, an IL-6 pathway inhibitor, has demonstrated efficacy in multiple studies in ameliorating orbital inflammation, diplopia, and proptosis, and is now recommended in the latest domestic and international clinical guidelines (2, 34). Concurrently, targeting the IL-17 pathway has emerged as a promising new frontier; however, its clinical application remains investigational. Preliminary evidence suggests that specific IL-17 antagonists—such as secukinumab—may confer therapeutic benefits. Future research should prioritize large-scale randomized controlled trials to definitively establish the efficacy and safety of IL-17 inhibition and to explore its potential role in personalized, precision-based management of TED.

The substantial heterogeneity observed across studies warrants careful consideration. Several factors may contribute to this variability. First, differences in thyroid function status—such as hyperthyroid, euthyroid, or hypothyroid states—may significantly influence systemic cytokine profiles, as thyroid hormones themselves modulate immune responses. Second, variations in disease stage (active vs. inactive) and treatment status likely affect circulating cytokine levels. Third, differences in detection methods, sample processing protocols, and data reporting formats may introduce methodological heterogeneity. Future studies should adopt standardized protocols, carefully document thyroid function status at the time of sampling, and report detailed clinical characteristics to facilitate more robust meta-analyses.

Subgroup analysis based on sample type revealed notable differences between serum and tear measurements. For IL-6, the pooled SMD was higher in serum (SMD: 1.90; 95% CI: 0.66–3.14) compared with tears (SMD: 1.19; 95% CI: 0.79–1.59). A similar pattern was observed for IL-17, with serum showing a larger effect size (SMD: 1.94; 95% CI: 1.08–2.80) than tears (SMD: 1.42; 95% CI: 0.74–2.10). These differences may reflect distinct pathophysiological compartments. Serum cytokine levels likely represent systemic inflammation, which may be influenced by thyroid function status, circulating autoantibodies, and systemic treatment. In contrast, tear cytokine levels may more directly reflect local orbital inflammation, including ocular surface involvement and lacrimal gland dysfunction. The wider confidence intervals observed in serum analyses also suggest greater variability, possibly due to differences in systemic treatment regimens and thyroid hormone levels across study populations. These findings highlight the importance of considering sample type when interpreting cytokine measurements in TED and suggest that serum and tears may provide complementary rather than interchangeable information.

Despite providing the quantitative synthesis of IL-6 and IL-17 levels in TED, this meta-analysis is subject to several limitations. First, the overall statistical power of the pooled results was limited, largely due to the predominance of small-scale studies, as large-sample, high-quality investigations remain scarce. Second, considerable heterogeneity was observed across the included studies, which may be explained by variations in detection methodologies, patient cohorts, and clinical definitions of disease activity and severity. Third, differences in treatment regimens and disease stages among the studies could have further confounded the estimates. Collectively, these limitations potentially bias the pooled results, underscoring the need for cautious interpretation and validation in future, well-designed prospective studies.

## Conclusion

To our knowledge, this meta-analysis is a large-scale systematic evaluation of IL-6 and IL-17 levels in patients with TED. The findings indicate elevated levels of both cytokines in TED patients compared with controls, with particularly high levels observed in active disease. However, the substantial heterogeneity across studies underscores the need for cautious interpretation. These findings describe the circulating cytokine profile in the studied population and may inform future hypothesis-driven research. Future high-quality, prospective studies with standardized detection methods and well-characterized patient cohorts are warranted to validate these findings and elucidate the underlying pathophysiological mechanisms.

## Data Availability

The original contributions presented in the study are included in the article/**Supplementary Material**. Further inquiries can be directed to the corresponding authors.

## Supplementary material

Supplementary File S1 Complete electronic search strategy.

Supplementary File S2 Preferred reporting items for systematic review and meta-analyses (PRISMA) checklist.

Supplementary Figure 1 The sensitivity analysis results of IL-6 in patients with thyroid eye disease compared to the control.

Supplementary Figure 2 The sensitivity analysis results of IL-17 in patients with thyroid eye disease compared to the control.

Supplementary Figure 3 The sensitivity analysis results of IL-6 in patients with active compared to the inactive thyroid eye disease.

Supplementary Figure 4 The sensitivity analysis results of IL-17 in patients with active compared to the inactive thyroid eye disease.

## References

[1] Wiersinga WM . Advances in treatment of active, moderate-to-severe Graves' ophthalmopathy. Lancet Diabetes Endocrinol. (2017) 5:134–42. doi: 10.1016/S2213-8587(16)30046-8. PMID: 27346786

[2] Bartalena L Kahaly GJ Baldeschi L Dayan CM Eckstein A Marcocci C . The 2021 European Group on Graves' orbitopathy (EUGOGO) clinical practice guidelines for the medical management of Graves' orbitopathy. Eur J Endocrinol. (2021) 185:G43–67. doi: 10.1530/EJE-21-0479. PMID: 34297684

[3] Ueland HO Neset MT Methlie P Ueland GA Pakdel F Rodahl E . Molecular biomarkers in thyroid eye disease: A literature review. Ophthalmic Plast Reconstr Surg. (2023) 39:S19–28. doi: 10.1097/IOP.0000000000002466. PMID: 38054982 PMC10697285

[4] Elia G Fallahi P Ragusa F Paparo SR Mazzi V Benvenga S . Precision medicine in Graves' disease and ophthalmopathy. Front Pharmacol. (2021) 12:754386. doi: 10.3389/fphar.2021.754386. PMID: 34776972 PMC8581657

[5] Murdock J Douglas RS Smith TJ . The role of IL-6 in thyroid eye disease: an update on emerging treatments. Front Ophthalmol (Lausanne). (2025) 4:1483578. doi: 10.3389/fopht.2025.1483578 PMC12034681 40297767

[6] Fallahi P Ferrari SM Elia G Ragusa F Paparo SR Patrizio A . Cytokines as targets of novel therapies for Graves' ophthalmopathy. Front Endocrinol (Lausanne). (2021) 12:654473. doi: 10.3389/fendo.2021.654473. PMID: 33935970 PMC8085526

[7] Wakelkamp IM Gerding MN Van Der Meer JW Prummel MF Wiersinga WM . Both Th1- and Th2-derived cytokines in serum are elevated in Graves' ophthalmopathy. Clin Exp Immunol. (2000) 121:453–7. doi: 10.1046/j.1365-2249.2000.01319.x. PMID: 10971510 PMC1905733

[8] Wei H Guan M Qin Y Xie C Fu X Gao F . Circulating levels of miR-146a and IL-17 are significantly correlated with the clinical activity of Graves' ophthalmopathy. Endocr J. (2014) 61:1087–92. doi: 10.1507/endocrj.EJ14-0267 25100151

[9] Laban-Guceva N Bogoev M Antova M . Serum concentrations of interleukin (IL-)1alpha, 1beta, 6 and tumor necrosis factor (TNF-) alpha in patients with thyroid eye disease (TED). Med Arh. (2007) 61:203–6. doi: 10.5455/medarh.2007.61.203-206. PMID: 18297990

[10] Riguetto CM Barbosa EB Atihe CC Reis F Alves M Zantut-Wittmann DE . Interaction of MMP-9 in the active phase of Graves' disease with and without ophthalmopathy. Am J Physiol Endocrinol Metab. (2024) 327:E577–84. doi: 10.1152/ajpendo.00126.2024 PMC11482230 39259164

[11] Wells GA Shea B O'Connell D Peterson J Welch V Losos M . The Newcastle-Ottawa Scale (NOS) for assessing the quality of nonrandomised studies in meta-analyses. Ottawa Hospital Research Institute (2014). Available online at: http://www.ohri.ca/programs/clinical_epidemiology/oxford.asp (Accessed December, 2025).

[12] Higgins JPT Green S . Cochrane handbook for systematic reviews of interventions version 5.1.0. The Cochrane Collaboration (2011). Available online at: http://www.cochrane-handbook.org (Accessed December, 2025).

[13] Wan X Wang W Liu J Tong T . Estimating the sample mean and standard deviation from the sample size, median, range and/or interquartile range. BMC Med Res Methodol. (2014) 14:135. doi: 10.1186/1471-2288-14-135. PMID: 25524443 PMC4383202

[14] Salvi M Pedrazzoni M Girasole G Giuliani N Minelli R Wall JR . Serum concentrations of proinflammatory cytokines in Graves' disease: effect of treatment, thyroid function, ophthalmopathy and cigarette smoking. Eur J Endocrinol. (2000) 143:197–202. doi: 10.1530/eje.0.1430197. PMID: 10913938

[15] Tang L Luo Q Zhou X Xia R . A study of cytokine expression in peripheral blood of patients with Graves' ophthalmopathy. Chin J Ophthalmol. (2002) 38:165–7. 11955323

[16] Kim SE Yoon JS Kim KH Lee SY . Increased serum interleukin-17 in Graves' ophthalmopathy. Graefes Arch Clin Exp Ophthalmol. (2012) 250:1521–6. doi: 10.1007/s00417-012-1995-7. PMID: 22752189

[17] Ujhelyi B Gogolak P Erdei A Nagy V Balazs E Rajnavolgyi E . Graves' orbitopathy results in profound changes in tear composition: a study of plasminogen activator inhibitor-1 and seven cytokines. Thyroid. (2012) 22:407–14. doi: 10.1089/thy.2011.0330. PMID: 22385289

[18] Lv M Shen J Li Z Zhao D Chen Z Wan H . Role of Treg/Th17 cells and related cytokines in Graves' ophthalmopathy. Nan Fang Yi Ke Da Xue Xue Bao. (2014) 34:1809–13. doi: 10.3969/j.issn.1673-4254.2014.12.21 25537908

[19] Shen J Li Z Li W Ge Y Xie M Lv M . Th1, Th2, and Th17 cytokine involvement in thyroid associated ophthalmopathy. Dis Markers. (2015) 2015:609593. doi: 10.1155/2015/609593. PMID: 26089587 PMC4451372

[20] Li W Wang JH . Comparison of tear cytokine levels in patients with thyroid-associated ophthalmopathy at different stages. Guangxi Med J. (2017) 39:812–4.

[21] Wang ZH . Significance of glucocorticoids in improving IL-17 and IL-21 levels and their correlation with efficacy in patients with active thyroid-associated ophthalmopathy. Int Eye Sci. (2017) 17:1643–5. doi: 10.3980/j.issn.1672-5123.2017.9.09

[22] Li HL Liu YQ Zheng ST Liu DZ Zhou L Yao D . Expression and clinical significance of serum IL-17 and IL-23 in patients with thyroid-associated ophthalmopathy. Chin J Mod Med. (2018) 28:105–8. doi: 10.1515/biol-2022-0694. PMID: 37671099 PMC10476477

[23] Zhang HJ Zhao M . Value of dynamic detection of serum IL-2 and IL-17 levels in assessing the condition of Graves' ophthalmopathy. Labeled Immunoassays Clin Med. (2019) 26:695–9.

[24] Xu N Cui Y Fu D Sun F . Tear inflammatory cytokines and ocular surface changes in patients with active thyroid eye disease treated with high-dose intravenous glucocorticoids. J Endocrinol Invest. (2020) 43:901–10. doi: 10.1007/s40618-019-01174-8. PMID: 31927748

[25] Kosciuszko M Poplawska-Kita A Pawlowski P Lipinska D Hryniewicka J Jankowska D . Clinical relevance of estimating circulating interleukin-17 and interleukin-23 during methylprednisolone therapy in Graves' orbitopathy: a preliminary study. Adv Med Sci. (2021) 66:315–20. doi: 10.1016/j.advms.2021.07.003. PMID: 34256242

[26] Mussakulova A Balmukhanova A Aubakirova A Zhunusova G Balmukhanova A Issakhanova J . Assessment of the levels of interleukin-17 and interleukin-38 in thyroid-associated ophthalmopathy patients. Int Ophthalmol. (2023) 43:2811–24. doi: 10.1007/s10792-023-02687-1. PMID: 36894821

[27] Huang JQ Chen Y Tong Y Qiu SL Wei W Zhang KP . Guiding value of serum IL-6, TNF-α, and sICAM-1 expression in evaluating the condition of patients with thyroid-associated ophthalmopathy. China Med Pharm. (2024) 14:143–7. doi: 10.1155/2022/2528046. PMID: 36419958 PMC9678454

[28] Zahir-Jouzdani F Atyabi F Mojtabavi N . Interleukin-6 participation in pathology of ocular diseases. Pathophysiology. (2017) 24:123–31. doi: 10.1016/j.pathophys.2017.05.001. PMID: 28629694

[29] Szabo-Fresnais N Blondeau JP Pomérance M . Activation of the cAMP pathway synergistically increases IL-1-induced IL-6 gene expression in FRTL-5 thyroid cells: involvement of AP-1 transcription factors. Mol Cell Endocrinol. (2008) 284:28–37. doi: 10.1016/j.mce.2008.01.013. PMID: 18280640

[30] Jyonouchi SC Valyasevi RW Harteneck DA Dutton CM Bahn RS . Interleukin-6 stimulates thyrotropin receptor expression in human orbital preadipocyte fibroblasts from patients with Graves' ophthalmopathy. Thyroid. (2001) 11:929–34. doi: 10.1089/105072501753211003. PMID: 11716039

[31] Hunt PJ Marshall SE Weetman AP Bell JI Wass JA Welsh KI . Cytokine gene polymorphisms in autoimmune thyroid disease. J Clin Endocrinol Metab. (2000) 85:1984–8. doi: 10.1210/jcem.85.5.6576. PMID: 10843185

[32] Korn T Bettelli E Oukka M Kuchroo VK . IL-17 and th17 cells. Annu Rev Immunol. (2009) 27:485–517. doi: 10.1146/annurev.immunol.021908.132710. PMID: 19132915

[33] Ruiz de Morales JMG Puig L Daudén E Cañete JD Pablos JL Olveira Martín A . Critical role of interleukin (IL)-17 in inflammatory and immune disorders: an updated review of the evidence focusing on controversies. Autoimmun Rev. (2020) 19:102429. doi: 10.1016/j.autrev.2019.102429. PMID: 31734402

[34] Burch HB Perros P Bednarczuk T Cooper DS Dolman PJ Leung AM . Management of thyroid eye disease: a consensus statement by the American Thyroid Association and the European Thyroid Association. Eur Thyroid J. (2022) 11:e220189. doi: 10.1530/ETJ-22-0189. PMID: 36479875 PMC9727317

