# Supplementary material for: Association of IL-6 and IL-17 with thyroid eye disease
Source: Front Endocrinol (Lausanne). 2026 May 8;17:1798278. doi: 10.3389/fendo.2026.1798278 (PMC13193857; doi:10.3389/fendo.2026.1798278)
Supplement: Supplementary File S1 — Complete electronic search strategy. [file DataSheet1.docx]

Complete electronic search strategy:

("Graves Ophthalmopathy"[Mesh] OR "Graves Orbitopathy"[Title/Abstract] OR "Thyroid Eye Disease"[Title/Abstract] OR "Thyroid Eye Diseases"[Title/Abstract] OR "Thyroid Associated Ophthalmopathy"[Title/Abstract] OR "Thyroid-Associated Ophthalmopathy"[Title/Abstract] OR "TAO"[Title/Abstract] OR "TED"[Title/Abstract] OR "Graves' Orbitopathy"[Title/Abstract] OR "Graves Orbitopathy"[Title/Abstract] OR "Ophthalmopathy, Graves"[Title/Abstract]) AND ("Interleukin-6"[Mesh] OR "Interleukin-6"[Title/Abstract] OR "IL-6"[Title/Abstract] OR "IL6"[Title/Abstract] OR "B-Cell Stimulatory Factor 2"[Title/Abstract] OR "Interleukin-17"[Mesh] OR "Interleukin-17"[Title/Abstract] OR "IL-17"[Title/Abstract] OR "IL17"[Title/Abstract] OR "CTLA-8"[Title/Abstract] OR "Cytotoxic T-Lymphocyte Associated Antigen 8"[Title/Abstract])
