# Supplementary figures and images for: Association of IL-6 and IL-17 with thyroid eye disease
Source: Front Endocrinol (Lausanne). 2026 May 8;17:1798278. doi: 10.3389/fendo.2026.1798278 (PMC13193857; doi:10.3389/fendo.2026.1798278)

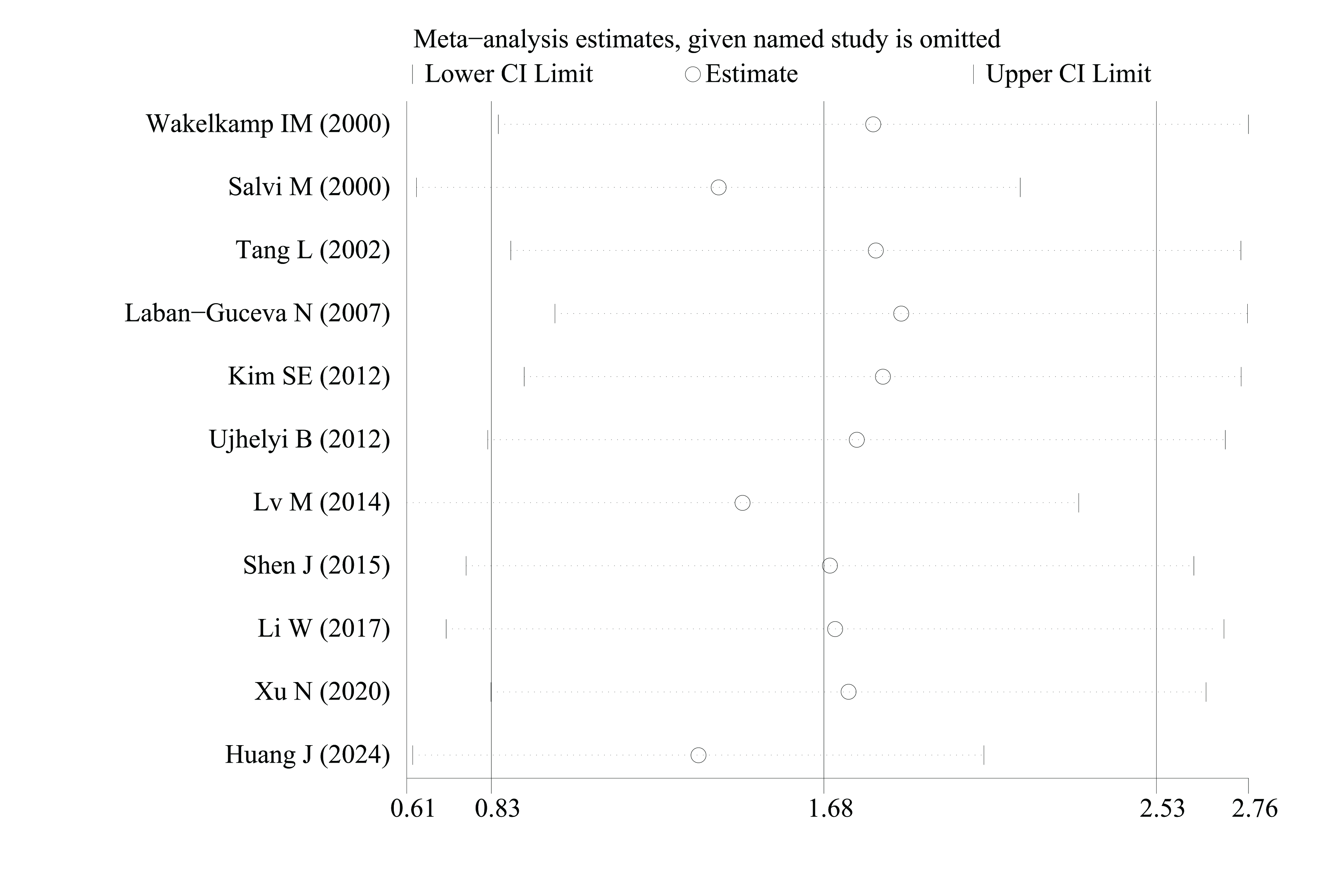

Supplement: Supplementary Figure 1 — The sensitivity analysis results of IL-6 in patients with thyroid eye disease compared to the control. [file Image1.tif]

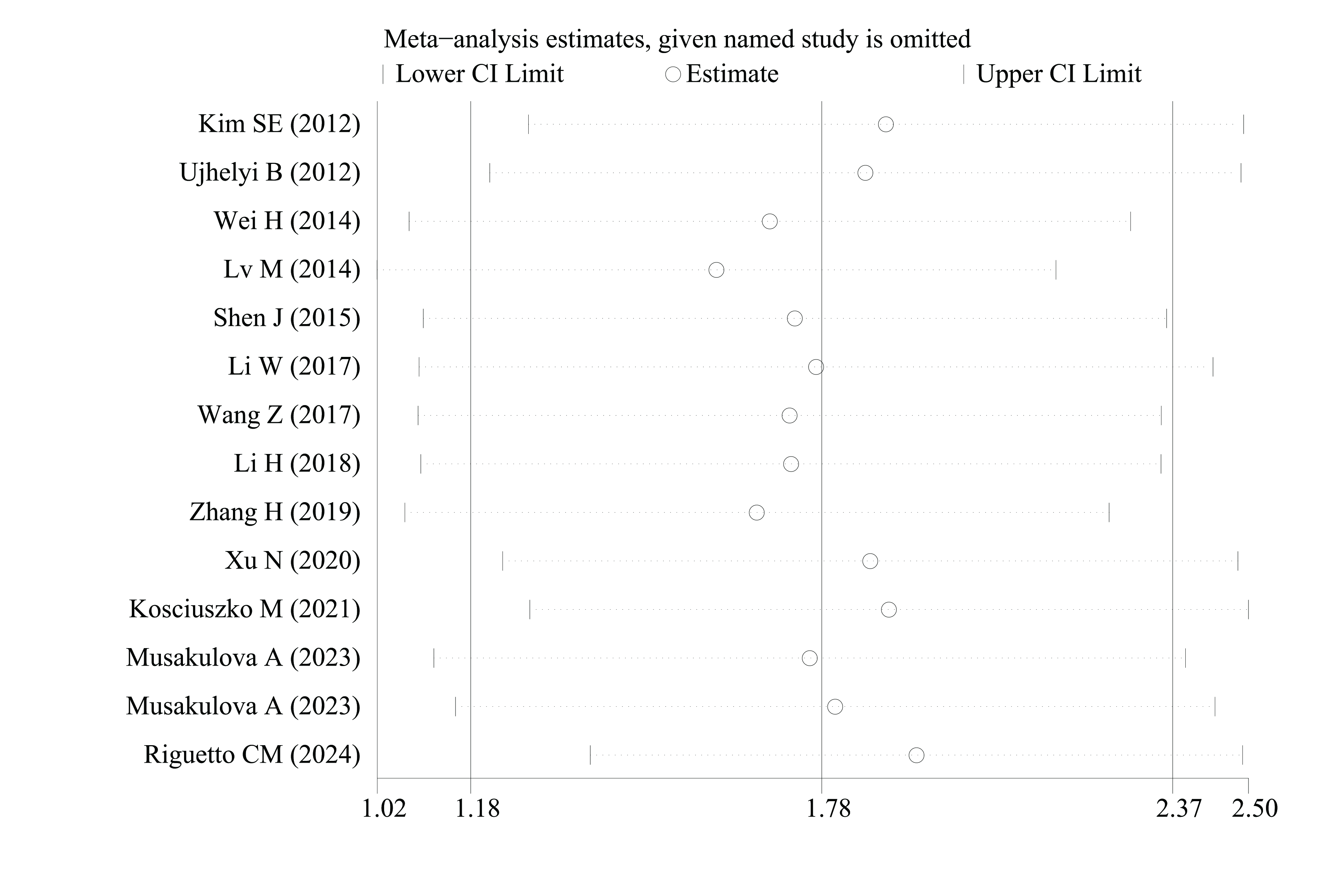

Supplement: Supplementary Figure 2 — The sensitivity analysis results of IL-17 in patients with thyroid eye disease compared to the control. [file Image2.tif]

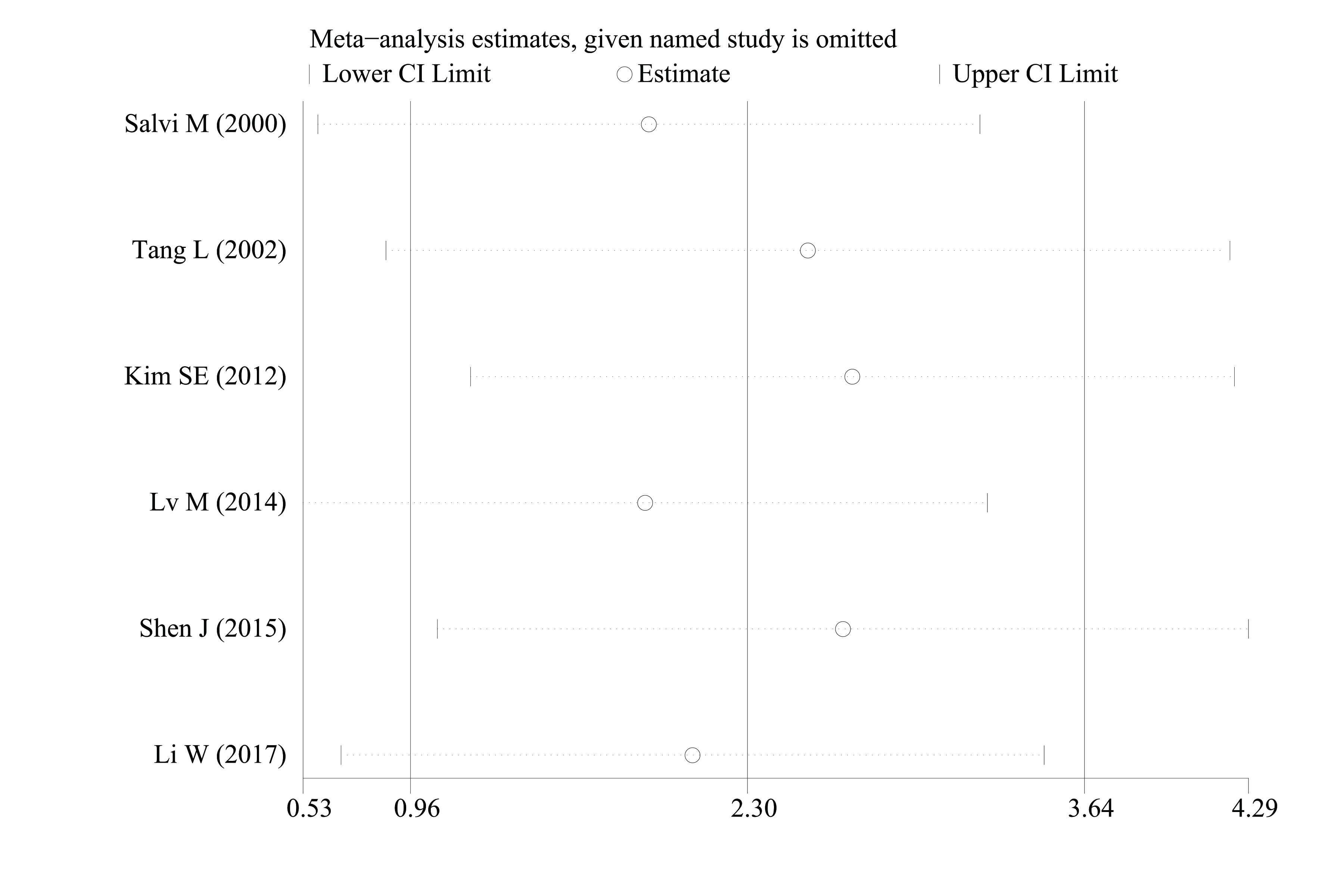

Supplement: Supplementary Figure 3 — The sensitivity analysis results of IL-6 in patients with active compared to the inactive thyroid eye disease. [file Image3.tif]

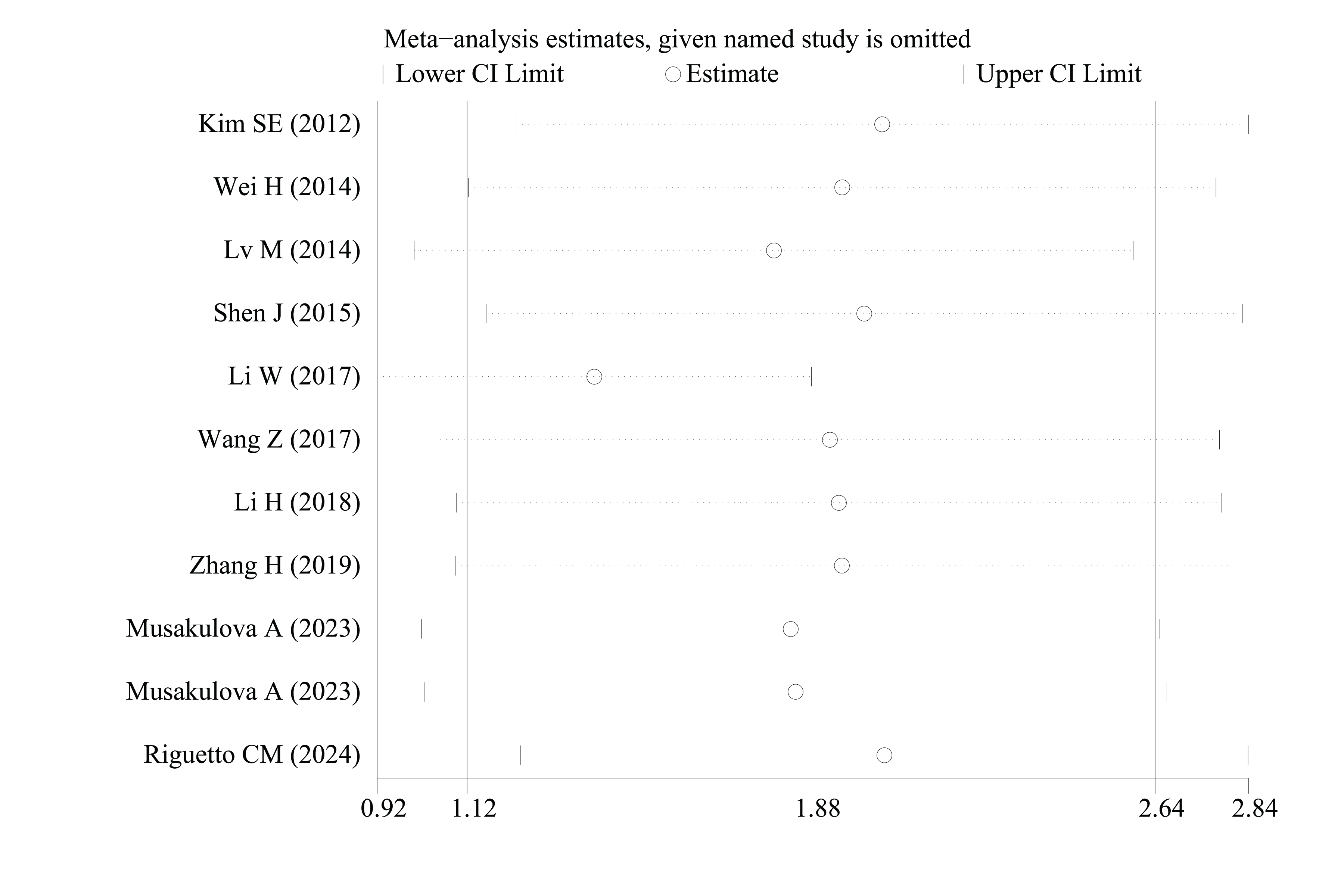

Supplement: Supplementary Figure 4 — The sensitivity analysis results of IL-17 in patients with active compared to the inactive thyroid eye disease. [file Image4.tif]
